# Neuroprotective efficacy of methanolic extract of propolis against early life stress-induced oxidative and inflammatory damage: Involvement of the Nrf2–Keap1 signaling pathway

**DOI:** 10.1016/j.ibneur.2025.10.010

**Published:** 2025-10-27

**Authors:** Ilia Ahmadi Kholardi, Akbar Hajizadeh Moghaddam, Mohammadreza Bigdeli, Sedigheh Khanjani Jelodar

**Affiliations:** a Department of Animal Science and Biotechnology, Faculty of Life Science and Biotechnology, Shahid Beheshti University, Tehran, Iran; b Department of Animal Science, Faculty of Science, University of Mazandaran, Babolsar, Iran; c Institute for Cognitive and Brain Science, Shahid Beheshti University, Tehran, Iran

**Keywords:** Methanolic extract of propolis, Early-life stress, Oxidative stress, Neuroinflammation

## Abstract

**Background:**

Early-life stress (ELS) is a major environmental risk factor for neurobehavioral impairments, including cognitive dysfunction, repetitive behaviors, and social deficits. Propolis, a natural bee product with strong antioxidant and anti-inflammatory properties, may counteract oxidative stress–related disorders. This study examined whether methanolic extract of propolis (MEP) mitigates ELS-induced oxidative and inflammatory damage in a maternal separation (MS) rat model, focusing on the Nrf2–Keap1 pathway.

**Methods:**

Male Wistar rats (200–220 g) were allocated to Control, Vehicle, Propolis (200 mg/kg), MS, and MS + Propolis (100 or 200 mg/kg) groups. ELS was induced by separating pups from dams for 3 h/day during postnatal days (P) 1–9. MEP was given orally from P21 to P42. Behavioral tests assessed social interaction, cognition, repetitive, and anxiety-like behaviors. Biochemical analyses measured antioxidant enzymes (SOD, CAT, GRx), glutathione, lipid peroxidation (MDA), and expression of Nrf2, Keap1, IL-6, and TNF-α.

**Results:**

MEP (100 and 200 mg/kg) improved social interaction (*p* < 0.05, *p* < 0.01), reduced repetitive behaviors (*p* < 0.001), and enhanced cognition (*p* < 0.05, *p* < 0.01) and anxiety-like behaviors (*p* < 0.001) in MS rats. Treatment increased SOD, CAT, and GRx activities (*p* < 0.01), elevated glutathione (*p* < 0.001), upregulated Nrf2 (*p* < 0.001), and reduced MDA (*p* < 0.001), IL-6, and TNF-α, with stronger effects at 200 mg/kg (*p* < 0.001).

**Conclusion:**

MEP alleviates oxidative and inflammatory responses induced by ELS, likely via Nrf2–Keap1 modulation, supporting its potential as a complementary therapy for stress-related neurobehavioral disorders.

## 1 Introduction

Appropriate environmental conditions during early development are critical for organizing functional patterns and morphological structures that persist throughout life. Exposure to stress during early life, particularly through maternal separation (MS) and early weaning, can have profound and long-lasting adverse effects on brain function, emotional regulation, and cognitive development (Nishi, 2020). Early-life stress (ELS) is strongly linked to a range of mental and behavioral abnormalities observed in both humans and animal models. Among these, MS is a well-established rodent model used to investigate the impact of ELS on emotional responses to challenges later in life (Zhang et al., 2024). This model also provides insights into how such responses may influence susceptibility or resilience to behavioral pathologies, including repetitive behaviors, cognitive deficits, impaired social interactions, and anxiety-like behaviors (Kambali et al., 2019, Cui et al., 2020, Farkas et al., 2009). Drawing on studies of modern societies and lifestyles, and in contrast to the outdated "refrigerator mother" theory, MS can be recognized as a significant societal issue with profound implications (Zeavin, 2021). Beyond its association with behavioral abnormalities resulting from ELS, maternal separation has been linked to reduced antioxidant enzyme activity and impaired neurogenesis in the hippocampus. Additionally, ELS has been shown to increase peripheral levels of pro-inflammatory cytokines, such as interleukin-6 (IL-6) and tumor necrosis factor-alpha (TNF-α) (Réus et al., 2017). The accumulation of oxidants disrupts cellular homeostasis by altering lipids, carbohydrates, and proteins, ultimately leading to cellular dysfunction. Under normal physiological conditions, a balance exists between the production of ROS and the cell's antioxidant capacity (Amini-Khoei et al., 2017). Alterations in the activities of enzymatic antioxidants, such as CAT, SOD, and GRx, as well as changes in detoxifying metabolites like GSH and markers of lipid peroxidation, have been observed in both the brain and peripheral systems of individuals exposed to ELS (Réus et al., 2017, Marković et al., 2017, Nouri et al., 2020). In immunological dysregulation and inflammation, mitochondrial dysfunction, oxidative stress, and the nuclear factor erythroid 2-related factor 2 (Nrf2) play critical roles. Under normal physiological conditions, Nrf2 interacts with Kelch-like ECH-associated protein 1 (Keap1) in the cytoplasm. However, during oxidative stress, Nrf2 dissociates from Keap1, translocates to the nucleus, and activates the antioxidant response element (ARE). This process leads to the upregulation of numerous genes involved in combating inflammatory and oxidative stress responses (Nadeem et al., 2020). The production of ROS is amplified by monocytes and leukocytes during inflammation, exacerbating tissue damage. In affected areas, the excessive release of ROS by inflammatory cells contributes to significant oxidative stress and cellular injury. Beyond this, ROS also play additional roles in inflammatory pathways, including the redox modulation of inflammatory mediators and transcriptional regulators linked to inflammatory responses (Bakunina et al., 2015). Extensive research has demonstrated that ELS upregulates the expression of pro-inflammatory cytokines. Numerous studies have identified significant changes in key pro-inflammatory cytokines, such as TNF-α and IL-6, which play critical roles in the development of behavioral abnormalities associated with ELS (Amini-Khoei et al., 2017, Hao et al., 2022). Numerous laboratory studies have demonstrated that ELS impacts critical brain regions, including the hypothalamic-pituitary-adrenal (HPA) axis, amygdala, cortex, and hippocampus (Wang et al., 2020). The hippocampus plays a vital role in cognitive function, language development, stress regulation, and memory formation. Given its significant growth during the early postnatal period, the hippocampus is highly vulnerable to external influences during this developmental stage. Extensive research has shown that MS impairs hippocampal function and induces oxidative stress and inflammatory responses (Zhang et al., 2024, Nouri et al., 2020, Malcon et al., 2020). Extending our prior research on maternal separation-induced apoptotic pathways in male rat cerebellum and hippocampus (Wang et al., 2020, Malcon et al., 2020), where we characterized the neuroprotective effects of quercetin and its nanophytosome formulation, the present study specifically investigates propolis-mediated modulation of hippocampal antioxidant and inflammatory signaling.

Propolis is a natural resinous compound synthesized by honey bees (*Apis mellifera*) from botanical sources. It exhibits a viscous texture and a distinctive spicy aroma, with colors ranging from green to dark brown. Notably, the physicochemical properties and biological activity of propolis exhibit substantial geographic variation, primarily determined by the diversity of local flora accessible to bee colonies (Fathi Hafshejani et al., 2023).

The biological activity of propolis is primarily attributed to its polyphenolic compounds, with flavonoids representing the most significant group of phenolic constituents. Flavonoids are widely used as a key criterion for evaluating the quality of propolis. These compounds exhibit remarkable properties, including anti-tumor, antioxidant, anti-inflammatory, neuroprotective, and various other bioactive effects (Mohammadzadeh et al., 2007, Tian et al., 2023, Mamashli et al., 2023). Notably, flavonoids demonstrate potent antioxidant and anti-inflammatory activities, which are closely linked to their concentration levels (Xu et al., 2022). Several studies have reported high levels of total phenolic and flavonoid content in Iranian propolis extracts (Asgharpour et al., 2020, Asgharpour et al., 2018, Salehi et al., 2022, Dadar et al., 2022). These chemical families are known to contribute to the antioxidant, anti-inflammatory, and neuroprotective effects of propolis. Recent findings support these neuroprotective properties; for example, Kherrab et al. reported that methanolic extract of propolis improved memory and preserved hippocampal and prefrontal cortical integrity in rats exposed to chronic mild stress during adolescence (Kherrab et al., 2024). One of the main mechanisms underlying these effects is the activation of the Nrf2/Keap1 signaling pathway, which regulates antioxidant response elements and cytoprotective gene expression. In silico molecular docking studies have further shown that flavonoid-rich propolis extracts can interact with key proteins in this pathway, suggesting a direct modulatory role (Reguig et al., 2025).

This study aimed to evaluate the therapeutic potential of *methanolic extract of propolis* (MEP) in mitigating behavioral abnormalities, oxidative stress, and neuroinflammation induced by maternal separation in male Wistar rats. Particular emphasis was placed on assessing its antioxidant and anti-inflammatory activities through modulation of the hippocampal Keap1/Nrf2 signaling pathway.

## 2 Materials and methods

### 2.1 Provision of propolis methanol extracts

Propolis samples were collected from honeybee colonies located in Mazandaran, Iran. To prepare the propolis extract, 50 g of powdered propolis was subjected to extraction with 500 mL of 80 % methanol (solvent-to-sample ratio 10 mL/g) for 24 h at room temperature (25°C) with occasional stirring. The resulting solution was filtered through double-layer coarse paper filters, and the solvent was evaporated using a rotary evaporator at 40°C. The concentrated extract was then freeze-dried and stored at −20°C for further use (Mohammadzadeh et al., 2007, Boulechfar et al., 2019, Shi et al., 2012).

### 2.2 GC/MS analysis

The chemical composition of MEP was characterized by gas chromatography-mass spectrometry (GC/MS) using an Agilent 6890 system (Agilent Technologies) equipped with an HP-5MS capillary column (30 m × 0.25 mm i.d., 0.2 μm film thickness). The temperature program initiated at 60°C (2 min hold), followed by a 5 °C/min ramp to 280°C (5 min hold). The injector temperature was maintained at 270°C with a 1:10 split ratio. Ultra-high purity helium carrier gas was delivered at a constant flow rate of 1.2 mL/min, with 1 μL sample injections performed in triplicate (Asghari et al., 2023, Hadavand Mirzaei et al., 2021). Phytochemical compounds were identified by comparing their retention times and mass spectra with established standards and quantified using calibration curves to elucidate the extract's chemical profile.

### 2.3 Experimental design

**Fig. 1:**
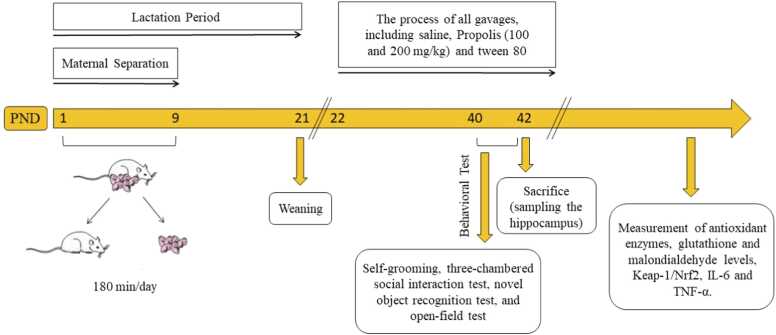
Scheme of the experimental method. Experimental timeline depicting maternal separation, propolis administration schedule, behavioral assessments, and hippocampal biochemical analyses.

All experimental procedures involving animals were approved by the Ethics Committee in accordance with the University of Mazandaran's guidelines for Animal Experimentation (IR.UMZ.REC.1400.020). For this study, adult female and male Wistar rats weighing between 200 and 250 g were selected and paired for mating. Only male offspring were used in the experiments to avoid the potential confounding effects of hormonal fluctuations across the estrous cycle in females, which can influence behavioral and biochemical parameters (Malcon et al., 2020, Asghari et al., 2023, Hadavand Mirzaei et al., 2021, Arabameri et al., 2017). Male offspring from each group were housed under standard laboratory conditions (22 ± 3 °C, 12-hour light/dark cycle) with ad libitum access to food and water. The male pups were divided into two main groups: control and maternal separation (MS). The control group was further subdivided into three subgroups: control (received saline orally), vehicle (received Tween 80 orally), and propolis (200 mg/kg, orally). Similarly, the MS group was subdivided into three subgroups: MS (received saline orally), propolis (100 mg/kg, orally), and propolis (200 mg/kg, orally). To induce maternal separation, pups in the MS groups were separated from their mothers daily from 9:00 AM to 12:00 PM and placed in plastic cages (20 × 20 cm) without woodchip bedding for three hours, starting from the first to the ninth postnatal day. From postnatal day 21 to day 42, all rats received daily oral gavage treatments, and sampling was performed on day 42. (Fig. 1) (Malcon et al., 2020, Arabameri et al., 2017).

### 2.4 Behavioral evaluation

#### 2.4.1 Grooming test

In the initial phase of the study, rats were acclimatized for five minutes in an open field apparatus measuring 60 × 60 cm. After a 24-hour interval, the animals were transferred to a specialized testing chamber for a 15-minute session to assess grooming behavior. The evaluation criteria included the total time spent on activities such as paw licking, nose and face cleaning, and scratching their fur with any limb. Following the assessment, the chamber was thoroughly sanitized using a 70 % ethanol solution to maintain a sterile environment for subsequent tests (Mansouri et al., 2021, Won et al., 2012).

#### 2.4.2 Social interaction test

Social behavior was assessed using a three-chamber apparatus. The test was conducted over a 15-minute period in a rectangular arena measuring 43 × 19 cm, divided into three sections by 22 cm high walls. Two wire-mesh compartments, each equipped with removable doors, were placed in the side chambers and contained a stranger mouse and a familiar mouse, respectively. Prior to testing, the rats were placed in the central empty chamber for 5 min to acclimate to the environment. On the day of the experiment, each rat's behavior was recorded using a digital camera for 10 min, and the time spent in each chamber was systematically quantified (Kaidanovich-Beilin et al., 2011).

#### 2.4.3 Novel object recognition test

The Novel Object Recognition Test (NORT) was conducted in three successive phases, habituation, acquaintance, and memory testing, over consecutive intervals. During the habituation phase, animals freely explored a designated enclosure. In the acquaintance phase, two identical objects (A+A) were introduced for interaction. Finally, the memory testing phase involved replacing one of the identical objects with a novel object (A+B). Exploration times for both familiar and novel objects were recorded and systematically analyzed to assess recognition capabilities (Hao et al., 2022, Yuede et al., 2009).

#### 2.4.4 Open field test

The behavioral assessment was conducted in a sound-attenuated testing room under standardized low illumination (≤50 lux). The apparatus consisted of a black polyvinyl chloride (PVC) square arena (40 × 40 × 40 cm) with floor markings dividing the surface into 16 equal quadrants (10 × 10 cm each). Animals were introduced directly into the arena without prior habituation. Locomotor activity was quantified over a 15-min test session using manual tracking with a digital chronometer, with total ambulatory distance (cm) recorded as the primary outcome measure (Mansouri et al., 2021).

### 2.5 Hippocampal tissue processing

Twenty-four hours following the final behavioral assessment, rats were euthanized and decapitated. The hippocampus was rapidly dissected bilaterally on an ice-cold platform, immediately snap-frozen in liquid nitrogen, and stored at −80°C until processing. For homogenization, tissue samples were suspended in ice-cold phosphate-buffered saline (PBS, 0.1 M, pH 7.4) and mechanically homogenized. The homogenate was centrifuged at 13,000 ×g for 30 min at 4°C. The resulting supernatant was aliquoted and stored at −80°C for subsequent biochemical analyses.

### 2.6 Catalase activity assay

Catalase (CAT) activity was determined spectrophotometrically according to the method of Aebi (1974). The reaction mixture (1 mL total volume) contained 10 mM H₂O₂ in 50 mM sodium phosphate buffer (pH 7.0) and 20 μL of tissue supernatant. The decomposition of H₂O₂ was monitored by measuring the decrease in absorbance at 240 nm (ε = 43.6 M⁻¹cm⁻¹) for 2 min at 25°C using a spectrophotometer (T80 + UV/VIS PG Instruments Ltd). A reference blank containing all reaction components except the enzyme extract was used for baseline correction.One unit of CAT activity was defined as the amount of enzyme required to degrade 1 μmol of H₂O₂ per minute per milligram of protein under the assay conditions.

### 2.7 Superoxide dismutase activity assay

Quantitative analysis of superoxide dismutase activity was performed utilizing the Genet et al. method, incorporating certain procedural modifications (Genet et al., 2002). The change in light absorption is measured at 420 nm for 4 min at 25°C compared to a blank solution containing all materials except homogeneous tissue. One unit of superoxide dismutase enzyme activity was determined by the amount of enzyme that inhibits half of the maximum Pyrogallol auto-oxidation.

### 2.8 Glutathione reductase activity assay

GRx activity was determined spectrophotometrically according to the method of Griffith (1980), with minor modifications. The assay mixture (800 μL final volume) contained: 100 mM potassium phosphate buffer (pH 7.0), 2.5 mM oxidized glutathione (GSSG) and 0.1 mM β-NADPH. The reaction was initiated by adding 60 μL of tissue homogenate supernatant to 740 μL of the pre-warmed (25°C) reaction mixture. NADPH oxidation was monitored continuously for 3 min at 340 nm (ε = 6.22 mM⁻¹cm⁻¹) using a spectrophotometer (T80 + UV/VIS PG instrument Ltd). Enzyme activity was calculated from the linear portion of the absorbance curve. One unit of GR activity was defined as the amount of enzyme required to oxidize 1 μmol of NADPH per minute per milligram of protein at 25°C.

### 2.9 Glutathione quantification

Total glutathione levels were determined using the established DTNB (5,5′-dithiobis-2-nitrobenzoic acid) assay as described by Ellman (1959). This colorimetric method quantifies sulfhydryl groups through the formation of 2-nitro-5-thiobenzoic acid (TNB), a yellow-colored chromophore with maximum absorbance at 412 nm. Briefly, reduced glutathione (GSH) reacts stoichiometrically with DTNB (1:1 molar ratio) under alkaline conditions (pH 8.0), producing the measurable TNB anion.

### 2.10 Quantification of malondialdehyde levels

MDA levels were determined using the methods outlined by Esterbauer and Cheeseman (1990). In this standardized protocol, MDA reacts with thiobarbituric acid (TBA) in a 1:2 molar ratio under acidic conditions (pH 2–3) at 95°C for 45 min, forming the MDA-TBA adduct. This chromogenic product exhibits maximum absorbance at 532–535 nm (ε = 1.56 × 10⁵ M⁻¹cm⁻¹), with the pink coloration intensity being proportional to MDA concentration. Spectrophotometric measurements were performed at 532 nm against a reagent blank containing all components except the sample.

### 2.11 Protein quantification

The quantification of the total protein content within each sample is conducted utilizing the Bradford colorimetric assay. This method is predicated on the protein-induced shift in the absorbance of Coomassie Brilliant Blue dye, facilitating a reliable measurement of protein concentration (Bradford, 1976).

### 2.12 Quantitative analysis of Keap1/Nrf2 and inflammatory cytokine expression

Gene expression levels of *Keap1*, *Nrf2*, *IL-6*, *TNF-α*, and reference gene *GAPDH* were analyzed by quantitative real-time PCR (qRT-PCR). Total RNA was isolated from hippocampal tissue using the RNeasy Micro Kit (Qiagen), followed by on-column DNase I (Fermentas) treatment to eliminate genomic DNA contamination. First-strand cDNA synthesis was performed using 1 μg total RNA with the RevertAid First Strand cDNA Synthesis Kit (Fermentas) according to the manufacturer's protocol. qRT-PCR amplification was conducted using SYBR Green Master Mix (TaKaRa). Relative gene expression was calculated using the comparative ΔΔCt method (Livak and Schmittgen, 2001, Sharma et al., 2016) with *GAPDH* as the endogenous control. Melting curve analysis confirmed amplification specificity( Table 2).

**Table2:** Sequences of primers used in qRT-PCR.

Gene	Primer	Sequence	Amplicon lengths (bp)
GAPDH	forward reverse	5 ´ -ATCCTGCACCACCAACTGC−3 ´ 5 ´ -ACGCCACAGCTTTCCAGAG−3 ´	129
Nrf2	forward reverse	5 ´ - TCAGCTACTCCCAGGTTGC−3 ´ 5 ´ - CAG GGC AAG CGA CTG AAA TG−3 ´	137
Keap1 TNF-α	Forward reverse forward reverse	5 ´ - TCG CAG GAT GGT AAC CGA AC−3 ´ 5 ´ - AAT TGG GCA GCT GGG ATG TC−3 5 ´ -GGAGGAGCAGCTGGAGTG−3 ´ 5 ´ -CCTTGAAGAGAACCTGGGAGTAGA−3 ´	146 131
IL−6	forward reverse	5 ´ -TCACAGAGGATACCACCCACAA−3 ´ 5 ´ -CAGTGCATCATCGCTGTTCATAC−3 ´	146

### 2.13 Statistical analysis

Obtained results indicated as mean ± SD and analyzed with one-way ANOVA and Tukey’s post-hoc test, as well as in all groups values were considered significant when *p* < 0.05.

## 3 Result

### 3.1 Chemical composition of propolis extract

GC-MS analysis identified and quantified several biologically active constituents in the MEP (Table 1). Chromanone emerged as the most abundant compound, representing (22.67 %) of the total extract composition. The flavonoid chrysin constituted the second major component (12.16 %), followed by pinostrobin chalcone (7.27 %). Additional quantitatively significant compounds included the flavonol galangin (6.75 %) and the anthraquinone derivative chrysophanol (6.33 %). These results demonstrate that chromanone, chrysin, and pinostrobin chalcone collectively represent the predominant phytochemical profile of the analyzed propolis extract.

**Table1:** GC-MS analysis of methanolic extract of Iranian propolis using HP-5MS capillary column and their percentages of area.

**Compounds**	**Area** **(%)**	**Quality** **(%)**
4-Chromanone	22.67	96
Chrysin	12.16	96
Pinostrobin chalcone	7.27	98
Galangin	6.75	89
Chrysophanol	6.33	59
5-Hydroxy−7-methoxy−6-methylflavone	5.84	98
Tricyclo[3.3.2.0(3,7)]decan−9-one	4.08	53
N-(2-Hydroxyphenyl)benzamide	3.89	35
Benzo[*h*]quinoline	3.05	59
3, 4-Dihydronaphthalene−2-carboxylic acid	2.25	91
trans-Ferulic Acid	2.09	70
N-(4-Methoxyphenyl)−2-hydroxyimino-acetamide	1.64	95
Genkwanin	1.60	72
2'-Hydroxy−5′-isopropyl−4′- ethylheptanophenone	1.53	50
Cinnamyl cinnamate	1.45	72
Guaiacylacetone	1.39	72
5-hydroxy−2-(4-hydroxyphenyl)−7-methoxy−2,3-dihydrochromen−4-one	1.28	89
4-methoxy benzaldehyde	1.23	52
2-methoxy−4- vinylphenol	1.12	95
3,4-Dimethoxycinnamic acid	1.09	97
Beta-EUDESMOL	0.85	96
Acacetin	0.81	53
Phenylethyl Alcohol	0.74	95
Benzyl Alcohol	0.73	97
N-(4-Methoxyphenyl)−2-hydroxyimino-acetamide	0.68	95
Benzoic acid	0.67	97
(*E*)−3-phenylprop−2-en−1-ol	0.66	98
(*E*)−3-phenyl−2-propenoic acid	0.64	98
1,4-diphenylbutane−1,4-dione	0.59	43
2,2′-Binaphthalene	0.58	38
2,3-dihydro−1-benzofuran	0.57	90
5-Methoxy−3,7-dihydroxyflavanone	0.56	62
Ethanedioic acid, [1,1′-biphenyl]−4-ylmethyl ethyl	0.48	47
2-Propenoic acid, 3-(4-hydroxyphenyl)-, methyl ester	0.48	53
Methyl hexadecanoate	0.48	96
(*E*)-octadec−9-enoic acid	0.45	99
Hexadecanoic acid	0.36	99
Alpha- EUDESMOL	0.33	98
Methyl (*Z*)-octadec−9-enoate	0.32	99
1-(4-Methoxymethyl−2,6-dimethylphenyl)ethanone	0.29	49

### 3.2 Effects of propolis on repetitive behaviors

**Fig. 2:**
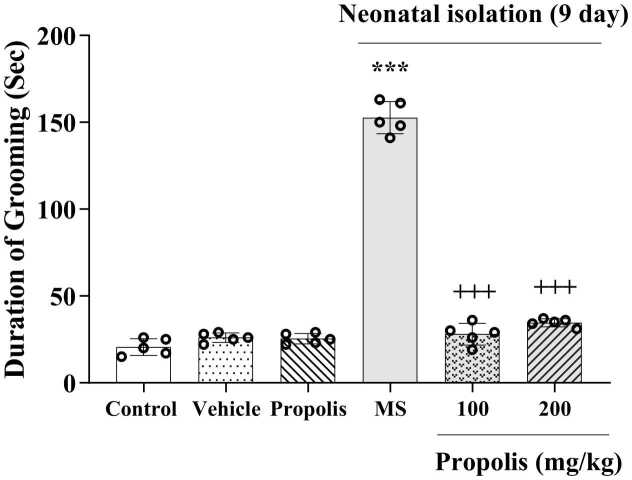
Effect of propolis on MS-induced repetitive behavior in grooming test. Animals were divided into five experimental groups: Control (saline-treated), Vehicle (Tween 80-treated), Propolis-only (200 mg/kg), Maternal separation (MS) and MS + propolis treatment (100 and 200 mg/kg). Data are presented as mean ± standard deviation (S.D.). Statistical comparisons were performed using Tukey's post hoc test following ANOVA. Significant differences are indicated as: ***p < 0.001 versus control group; + ++*p* < 0.001 versus MS group.

The MS paradigm induced a significant increase in repetitive grooming behaviors compared to non-separated controls (*p* < 0.001). Notably, both doses of MEP (100 and 200 mg/kg) effectively attenuated these MS-induced repetitive behaviors, showing statistically significant reductions versus the MS-only group (*p* < 0.001; Fig. 2). The reduction was significant at both dosages of propolis compared to the MS group, and also not significantly different from the control group. indicating that treatment restored this parameter to near-control levels.

### 3.3 Effects of propolis on social interaction behavior

**Fig. 3:**
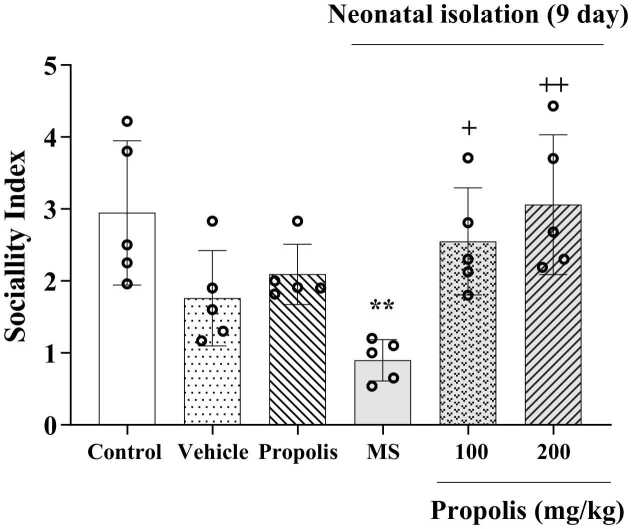
Effect of propolis on MS-induced sociality index in the social test. Animals were divided into five experimental groups: Control (saline-treated), Vehicle (Tween 80-treated), Propolis-only (200 mg/kg), Maternal separation (MS) and MS + propolis treatment (100 and 200 mg/kg). Data are presented as mean ± standard deviation (S.D.). Statistical comparisons were performed using Tukey's post hoc test following ANOVA. Significant differences are indicated as: ** *p* < 0.01, vs. control group, + *p* < 0.05, + + *p* < 0.01, vs. MS grou*p*.

Quantitative analysis of social interaction behaviors demonstrated significant group differences (Fig. 3). Rats subjected to maternal separation exhibited markedly reduced social interaction compared to control animals (*p* < 0.01). Notably, propolis administration at both 100 mg/kg (*p* < 0.05) and 200 mg/kg (*p* < 0.01) doses significantly ameliorated these social deficits, with the higher dose showing greater efficacy in restoring normal social interaction patterns. Propolis at 200 mg/kg showed no significant difference from controls, whereas Propolis100 remained slightly lower than controls.

### 3.4 Effects of propolis on cognitive function

**Fig. 4:**
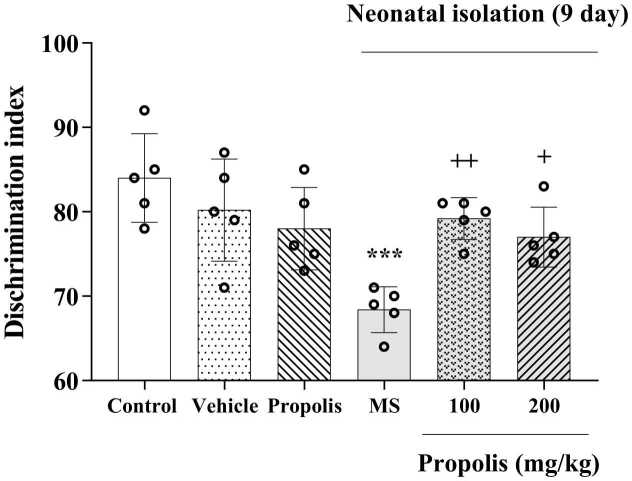
Effect of propolis on MS-induced cognitive deficits in the NORT. Animals were divided into five experimental groups: Control (saline-treated), Vehicle (Tween 80-treated), Propolis-only (200 mg/kg), Maternal separation (MS) and MS + propolis treatment (100 and 200 mg/kg). Data are presented as mean ± standard deviation (S.D.). Statistical comparisons were performed using Tukey's post hoc test following ANOVA. Significant differences are indicated as: *** *p* < 0.001, vs. control group, + + *p* < 0.01, + *p* < 0.05, vs. MS grou*p*.

The NORT revealed significant cognitive impairment in MS rats, as evidenced by a markedly reduced discrimination index compared with the non-separated control group (*p* < 0.001). Propolis treatment demonstrated dose-dependent neuroprotective effects, with both 100 mg/kg (*p* < 0.05) and 200 mg/kg (*p* < 0.01) doses significantly improving the discrimination index versus the MS-only group (Fig. 4). The discrimination index in the 200 mg/kg group was not significantly different from controls, whereas the 100 mg/kg group remained slightly lower than controls.

### 3.5 Effects of propolis on anxiety-like behaviors

**Fig. 5:**
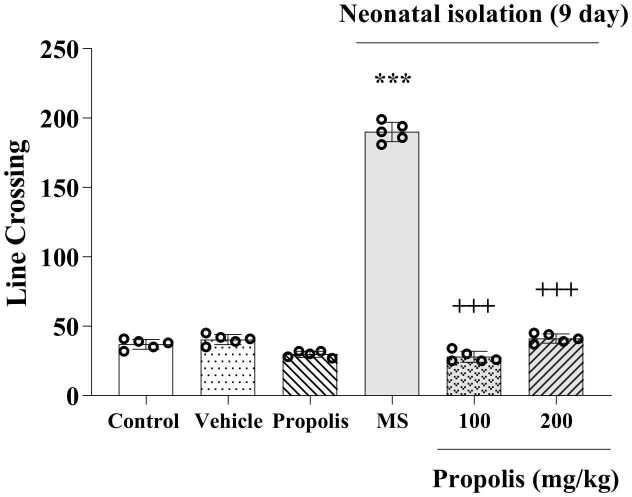
Effect of propolis on MS-induced anxiety-like behaviors in the OFT. Animals were divided into five experimental groups: Control (saline-treated), Vehicle (Tween 80-treated), Propolis-only (200 mg/kg), Maternal separation (MS) and MS + propolis treatment (100 and 200 mg/kg). Data are presented as mean ± standard deviation (S.D.). Statistical comparisons were performed using Tukey's post hoc test following ANOVA. Significant differences are indicated as: *** *p* < 0.001 vs. control group; + ++ *p* < 0.001 vs. MS group.

Maternal separation markedly increased locomotor activity, as indicated by a higher number of line crossings compared with the control group (p < 0.001; Fig. 5). Both 100 and 200 mg/kg doses of propolis significantly reduced this MS-induced hyperlocomotion (p < 0.001 vs. MS), restoring locomotor activity toward control levels. No significant differences were observed among the control, vehicle, and non-stressed propolis groups.

### 3.6 Effects of propolis on hippocampal MDA and GSH levels

Following the assessment of MDA and GSH levels, the MS group exhibited a significant elevation in MDA levels compared with the non-separated control group (*p* < 0.001). Propolis treatment at both 100 and 200 mg/kg markedly attenuated this increase, showing significant reductions versus the MS-only group (*p* < 0.001). MDA levels in the 200 mg/kg group were comparable to controls, whereas the 100 mg/kg group showed a partial reduction without full normalization. In contrast, GSH levels were significantly lower in the MS group than in controls (*p* < 0.001). Propolis administration at both doses effectively restored GSH levels, resulting in significant increases versus the MS group (*p* < 0.001), with the 200 mg/kg group reaching values similar to controls and the 100 mg/kg group showing near-complete recovery (Tables 3).

**Table3:** Effect of Propolis on MDA and GSH levels in Hippocampus.

Groups	MDA (µg/mg protein)	GSH (mg/gr protein)
Control	0.21 ± 0.03	0.54 ± 0.04
Vehicle	0.26 ± 0.01	0.50 ± 0.03
Propolis	0.23 ± 0.03	0.62 ± 0.01
MS	0.85 ± 0.04^***^	0.10 ± 0.01^***^
Propolis 100	0.34 ± 0.01^+++^	0.60 ± 0.03^+++^
Propolis 200	0.20 ± 0.02^+++^	0.57 ± 0.01^+++^

Data are reported as the mean ± SD of five rats in each group. MS means maternal separation. Tukey's Post hoc test was used to compare between groups. *** *p* < 0.001 as compared to the control group. + ++ *p* < 0.001 as compared to MS group.

### 3.7 Effects of propolis on hippocampal CAT, SOD, and GRx enzymes activity

The MS group showed a significant reduction in the activity of antioxidant enzymes (CAT, SOD, and GRx) compared with the non-separated control group (*P* < 0.001). Propolis treatment at both 100 and 200 mg/kg effectively restored the activity of these enzymes, showing significant increases versus the MS-only group (*P* < 0.01; Table 4). Enzyme activities in the 200 mg/kg group were comparable to controls, whereas the 100 mg/kg group showed partial recovery without full normalization.

**Table4:** Effect of Propolis on the activity of Hippocampal antioxidant enzymes.

Groups	CAT (U/mg protein)	SOD (% inhibition)	GRx (U/mg protein)
Control	133.59 ± 12.66	95.46 ± 1.76	124.02 ± 9.32
Vehicle	135.22 ± 4.90	89.76 ± 1.43	127.42 ± 2.41
Propolis	167.32 ± 14.98	93.88 ± 1.10	143.30 ± 12.08
MS	61.03 ± 7.68^***^	63.23 ± 1.77^***^	47.09 ± 5.21^***^
Propolis 100	117.51 ± 9.08^++^	83.75 ± 2.31^+++^	107.36 ± 3.27^+++^
Propolis 200	155.91 ± 7.75^+++^	93.72 ± 1.89^+++^	99.19 ± 2.30^+++^

Data are reported as the mean ± SD of five rats in each group. MS means maternal separation. Tukey's Post hoc test was used to compare between groups. *** *p* < 0.001 as compared to the control group. + + *p* < 0.01, + ++ *p* < 0.001 as compared to MS group.

### 3.8 Effects of propolis on the expression of hippocampal Nrf2 and keap1 genes

**Fig. 6:**
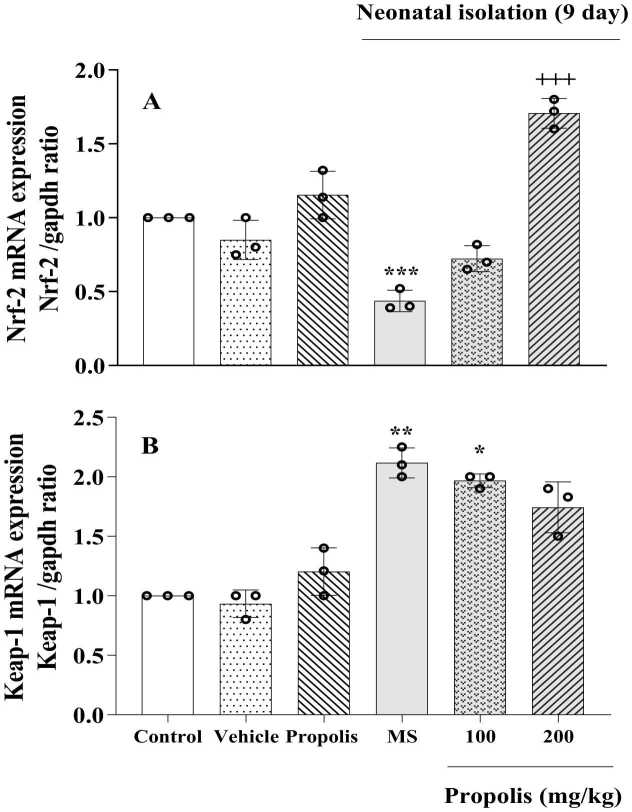
Effect of propolis on hippocampal Nrf2 and keap-1 expressions in MS rats. Animals were divided into five experimental groups: Control (saline-treated), Vehicle (Tween 80-treated), Propolis-only (200 mg/kg), Maternal separation (MS) and MS + propolis treatment (100 and 200 mg/kg). Data are presented as mean ± standard deviation (S.D.). Statistical comparisons were performed using Tukey's post hoc test following ANOVA. Significant differences are indicated as: * *p* < 0.05, ** *p* < 0.01 and *** *p* < 0.001 vs. control group; + ++ *p* < 0.001 vs. MS group.

Our findings revealed that Keap1 expression was significantly upregulated in the MS group compared with the non-separated control group (*p* < 0.01). However, treatment with propolis at 200 mg/kg did not produce a statistically significant change in Keap1 expression compared with the MS-only group, and expression levels remained elevated relative to controls. Similarly, the 100 mg/kg dose did not significantly alter Keap1 expression versus the MS group, with values still higher than controls. In contrast, Nrf2 expression was markedly downregulated in the MS group compared with controls (*p* < 0.001). Propolis administration at 200 mg/kg significantly restored Nrf2 expression, showing a robust increase versus the MS-only group (*p* < 0.001), with expression levels closely approximating those observed in the control group. The 100 mg/kg dose also increased Nrf2 expression compared with the MS group, but the improvement was less pronounced and did not fully reach control levels (Fig. 6).

### 3.9 Effects of propolis on hippocampal IL-6 and TNF-α gene expression

**Fig. 7:**
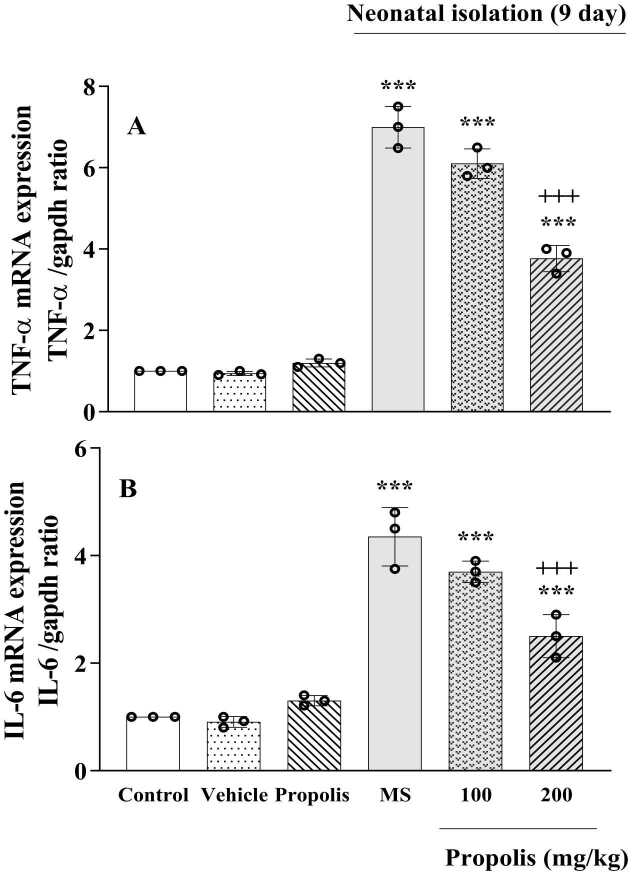
Effect of propolis on TNF-α and Il-6 expressions in MS rats. Animals were divided into five experimental groups: Control (saline-treated), Vehicle (Tween 80-treated), Propolis-only (200 mg/kg), Maternal separation (MS) and MS + propolis treatment (100 and 200 mg/kg). Data are presented as mean ± standard deviation (S.D.). Statistical comparisons were performed using Tukey's post hoc test following ANOVA. Significant differences are indicated as: *** *p* < 0.001 vs. control group. + ++ *p* < 0.001 vs. MS group.

Our results demonstrated that MS induction led to significant upregulation of pro-inflammatory cytokine genes, with both IL-6 and TNF-α showing markedly increased expression compared with the non-separated control group (***p*** < 0.001). Propolis administration at 200 mg/kg effectively attenuated this inflammatory response, significantly downregulating the expression of both IL-6 and TNF-α versus the MS-only group (*p* < 0.001). The 100 mg/kg dose also reduced IL-6 and TNF-α expression compared with the MS group, although the magnitude of change was smaller than that observed with 200 mg/kg, and expression levels in the 100 mg/kg group remained slightly above control values (Fig. 7).

## 4 Discussion

The primary objective of this study was to investigate whether methanolic extract of propolis (MEP) could mitigate maternal separation–induced behavioral abnormalities, oxidative stress, and neuroinflammation in male Wistar rats, with a particular focus on the potential modulation of the hippocampal Keap1/Nrf2 signaling pathway.

Our findings demonstrate that maternal separation (MS) significantly reduces social interaction while exacerbating cognitive impairment, repetitive behaviors, and anxiety-like symptoms. These results are consistent with previous reports showing that early life stress (ELS) in rodents induces similar behavioral alterations. Notably, existing literature corroborates our observations that MS leads to depressive-like symptoms, increased self-grooming, anxiety behaviors, and impaired social interactions - all indicative of underlying neuronal dysfunction (Hao et al., 2022, Mansouri et al., 2021, Wu et al., 2014, Harrison et al., 2014). ELS increases environmental oxidant exposure in neonatal rats, resulting in elevated ROS production. These detrimental effects are mediated primarily through the disruption of antioxidant enzyme systems (Malcon et al., 2020). Our results demonstrate that MS induction significantly elevated hippocampal MDA levels while reducing GSH content and diminishing GRx, CAT, and SOD enzymatic activity. These findings indicate that oxidative stress-mediated damage extends beyond lipid peroxidation to include membrane protein degradation. This observation aligns with previous reports showing that ELS induced by the MS model elevates MDA levels (Nouri et al., 2020), depletes GSH (Amini-Khoei et al., 2017), and reduces SOD and CAT activity (Réus et al., 2017). Notably, propolis has been widely recognized as a potent antioxidant with significant free radical scavenging capacity in multiple experimental models (Mohammadzadeh et al., 2007, Tian et al., 2023, Wieczorek et al., 2022). GC-Mass analysis of the MEP identified multiple bioactive compounds exhibiting potent antioxidant and anti-inflammatory activities. Notably, chromanone and chrysin demonstrated dual protective mechanisms: (1) direct free radical scavenging and oxidative stress reduction, and (2) significant anti-inflammatory effects mediated through downregulation of pro-inflammatory cytokine expression (IL-1β, TNF-α) and suppression of NF-κB signaling pathways. These coordinated actions contribute to enhanced cellular protection and homeostasis maintenance (Kamboj and Singh, 2022, Çelik et al., 2020, Li et al., 2023, Li et al., 2022). The synergistic effects of these compounds underscore the multifaceted therapeutic potential of propolis extract. The varying concentrations of its bioactive components reflect the extract's complexity, indicating that its pharmacological properties stem from their combined and cooperative actions—particularly in antioxidant and anti-inflammatory effects. Our research findings indicate that propolis ameliorates MS-induced behavior impairments, including improvements in self-grooming, social interaction, cognitive deficits, and anxiety-like behaviors. Additionally, propolis upregulates key antioxidant indexes **(**GSH, CAT, GRx, and SOD) while reducing MDA production. To validate these findings, subsequent studies examined propolis's capacity to elevate antioxidant activity, attenuate lipid peroxidation, and boost GSH levels in male rat models (Kocot et al., 2018, Yousef and Salama, 2009, Abdel-Rahman et al., 2020). Elevated ROS production triggers astrocyte and microglial activation, resulting in increased secretion of pro-inflammatory chemokines and cytokines (Rancan et al., 2018). Altered cytokine activity has been demonstrated to increase blood-brain barrier permeability, contributing to neural damage as observed in ELS models (Réus et al., 2017, Banks, 2008). Our results indicate that ELS induction via the MS paradigm significantly upregulated hippocampal expression of pro-inflammatory cytokine genes (IL-6, TNF-α). These findings suggest that MS exposure initiates inflammatory cytokine release, subsequently inducing hippocampal neuroinflammation and structural compromise (Wang et al., 2020, Xie et al., 2017).

A primary objective of this study was to examine propolis-mediated modulation of IL-6 and TNF-α gene expression. Our results revealed that high-dose propolis administration significantly attenuated inflammatory gene expression in the hippocampus of MS model animals. These findings align with previous work by Wu et al., who reported that propolis (50 μg/mL) exhibits anti-inflammatory properties in Alzheimer's disease models (Aabed et al., 2019, Wu et al., 2017). Furthermore, Askari et al.'s findings provide additional validation, demonstrating that propolis administration (100–200 mg/kg) effectively downregulates pro-inflammatory cytokine expression, including IL-6 and TNF-α (Askari et al., 2018). The Keap1-Nrf2 pathway represents a pivotal regulatory mechanism for cellular defense against oxidative stress. Under oxidative stress conditions, Nrf2 dissociates from Keap1 and translocates to the nucleus, initiating transcription of antioxidant response element (ARE)-driven genes that encode cytoprotective enzymes (Bertrand et al., 2015). In chronic neurological disorders, Nrf2 signaling is often impaired, resulting in compromised antioxidant defenses. This has positioned the Keap1-Nrf2 pathway as a promising therapeutic target for conditions characterized by oxidative damage and neuroinflammation (Ramsey et al., 2007, Li et al., 2020).

The present study demonstrates that MS significantly downregulates Nrf2 gene expression. Our findings reveal a dose-dependent antioxidant effect of propolis, with higher doses markedly upregulating Nrf2 expression in the hippocampus. This upregulation correlates with attenuated oxidative damage in brain tissue. Existing evidence links ELS to mitochondrial dysfunction, exacerbated oxidative stress, and impaired Nrf2 signaling (Hoffmann and Spengler, 2018). Mechanistically, propolis appears to enhance Nrf2 expression via its potent antioxidant activity (Tian et al., 2023, Xu et al., 2022, Cardinault et al., 2020). Barroso et al. further demonstrated that propolis may interfere with Keap1-Nrf2 binding, thereby suppressing inflammatory cascades while promoting Nrf2 activation (Barroso et al., 2017). Collectively, these effects contribute to reduced neuroinflammation and oxidative damage.

Although the behavioral and biochemical assays used are widely accepted, inherent variability in these methods may influence the precision of the results, particularly in complex neurobehavioral domains. To address this limitation, future studies should incorporate compound-specific approaches, focusing on dominant bioactive constituents such as chromanones identified in our extract. Investigating their isolated effects on complementary signaling pathways including NF-κB, BDNF, and MAPK may provide a more mechanistic and reproducible understanding of propolis-mediated neuroprotection.

## 5 Conclusion

This study provides compelling evidence that methanolic extract of Iranian propolis (MEP) alleviates behavioral and biochemical abnormalities induced by early-life stress, primarily through its dual antioxidant and anti-inflammatory actions. The observed upregulation of the hippocampal Nrf2-Keap1 pathway and suppression of pro-inflammatory cytokines suggest a mechanistic basis for its neuroprotective efficacy. These findings support the potential of propolis as a natural adjunctive agent for mitigating stress-related neuropsychiatric conditions. Based on this evidence, we recommend further investigation into its clinical applicability, including formulation optimization, long-term safety profiling, and integration with existing therapeutic regimens. Future studies should also explore sex-specific responses, broader dose ranges, and complementary molecular pathways to fully characterize its mechanistic landscape and translational potential.

## CRediT authorship contribution statement

**Akbar Hajizadeh Moghaddam:** Writing – review & editing, Writing – original draft, Project administration, Formal analysis, Conceptualization. **Ilia Ahmadi Kholardi:** Writing – original draft, Project administration, Investigation, Data curation. **Sedigheh Khanjani Jelodar:** Writing – original draft, Methodology, Formal analysis, Conceptualization. **Mohammadreza Bigdeli:** Visualization, Resources, Conceptualization.

## Ethical standards

Experimental protocols were based on the ethical rules approved by the University of Mazandaran Ethics Committee ((IR.UMZ.REC.1400.020)). All chemicals utilized in the present investigation were purchased from Sigma-Aldrich, Alfasan, and Merck.

## Declaration of Generative AI and AI-assisted technologies in the writing process

**Statement:** During the preparation of this work the author(s) used [deepseek] in order to [Enghlish gramer cheke]. After using this tool/service, the author(s) reviewed and edited the content as needed and take(s) full responsibility for the content of the published article.

## Funding

This research did not receive any specific grant from funding agencies in the public, commercial, or not-for-profit sectors.

## Conflicts of Interest

The authors declare that they have no known competing financial and personal relationships with other individuals or institutions that could affect their investigation reported in this paper.
